# The systemic inflammation indexes predict all-cause mortality in peritoneal dialysis patients

**DOI:** 10.1080/0886022X.2022.2160348

**Published:** 2023-07-07

**Authors:** Yan Yang, Yuanyuan Xu, Shusu Liu, Peiyu Lu, Hua Zhou, Min Yang

**Affiliations:** Department of Nephrology, The Third Affiliated Hospital of Soochow University, Changzhou, China

**Keywords:** Aggregate index of systemic inflammation, systemic immune-inflammation index, systemic inflammation response index, peritoneal dialysis

## Abstract

**Background:**

Chronic inflammation is a common complication in peritoneal dialysis (PD) patients. The aim of this study is to investigate the capacity of aggregate index of systemic inflammation (AISI), systemic immune-inflammation index (SII), and systemic inflammation response index (SIRI) to predict all-cause mortality in PD patients.

**Methods:**

This was a single-center retrospective study. The optimal cutoff values were identified by receiver operating characteristic (ROC) curve analysis. The area under the curve (AUC) was calculated to evaluate the predictive ability of these indexes. The Kaplan–Meier curves and log-rank test were performed to estimate cumulative survival rate. Cox proportional hazards regression analyses were conducted to determine the independent prognostic power of inflammation indexes.

**Results:**

A total of 369 incident PD patients were involved. During a median follow-up period of 32.83 months, 65 patients (24.2%) died. The ROC analysis indicated the largest value of AUC was obtained for SII (AUC = 0.644, 95% CI = 0.573–0.715, *p* < .001), followed in order by AISI (AUC = 0.617, 95% CI = 0.541–0.693, *p* = .003), and SIRI (AUC = 0.612, 95% CI = 0.535–0.688, *p*  = .004). The Kaplan–Meier survival curves revealed significantly lower survival rate with higher AISI (*p*  = .001), higher SSI (*p*  = .001), and higher SIRI (*p*  = .003). Even after adjustment for the confounding factors, higher AISI [hazard ratio (HR)=2.508, 95% confidence intervals (CI)=1.505–4.179, *p* < .001), SII (HR = 3.477, 95% CI = 1.785–6.775, *p* < .001), and SIRI (HR = 1.711, 95% CI = 1.012–2.895, *p*  = .045) remained as independent predictors of all-cause mortality.

**Conclusions:**

The higher AISI, SII, and SIRI were independent indicators of all-cause mortality in PD patients. Furthermore, they could provide comparable predictive value and assist clinicians to ameliorate PD management.

## Introduction

Kidney failure (KF) patients are confronted with chronic inflammation that attenuates life span and quality of life. Compared with hemodialysis (HD), peritoneal dialysis (PD) has advantages of better-preserved residual kidney function (RKF), improved patient survival, higher patient satisfaction and reduced financial stress to governments [1]. Despite all this, the mortality is 6.1 to 7.8 times higher than age-matched general population [2]. Among PD patients, inflammation is a critical component of ‘malnutrition-inflammation complex syndrome’ and a powerful predictor of mortality [3,4]. The presence of inflammation in PD is often noticed by increased levels of inflammatory mediators, such as C-reactive protein (CRP) and interleukin-6 (IL-6) [5]. On the basis of the different cutoff values of CRP, the estimated incidence of systemic inflammation ranged from 12% to 65% in PD patients [6]. Increased CRP was confirmed as an independent predictor of all-cause and cardiovascular mortality in PD patients [7]. Subsequently, the prognostic values of CRP and IL-6 have been proven in numerous studies [8].

In recent years, blood cell count-derived inflammation indexes have attracted more attentions because of inexpensive cost, simple assay and easy calculation. Previous researches demonstrated that neutrophil to lymphocyte ratio (NLR) [9], platelet to lymphocyte ratio (PLR) [10] and monocyte to lymphocyte ratio (MLR) [11] were significantly and independently associated with mortality in PD patients. They are suitable for routine use and regarded as reproducible indicators of the systemic inflammation response. Recently, Li et al. found systemic inflammation response index (SIRI) was a promising predictor of all-cause mortality and cardiovascular mortality in PD patients [12]. However, there are no studies evaluating prognostic values of aggregate index of systemic inflammation (AISI), and systemic immune-inflammation index (SII). As a consequence, in the present study, we aimed to investigate and compare the capacity of SIRI, AISI, and SII to predict all-cause mortality in PD patients.

## Materials and methods

### Study design and population

We retrospectively studied PD patients admitted to the Department of Nephrology of the Third Affiliated Hospital of Soochow University between January 2010 and December 2019. The exclusion criteria were as followed: (1) younger than 18 years old; (2) diagnosis with malignancies, hematological diseases, autoimmune diseases, severe infection or liver failure; (3) receiving immunosuppression therapy within 6 months; (4) maintenance PD therapy less than 3 months, lost to follow-up or transferred to other PD centers. All patients signed informed consent at the commencement of PD. The study was in line with the principles of the Declaration of Helsinki and with approved by the Ethics Committee at the Third Affiliated Hospital of Soochow University, China.

### Data collection and follow-up

Baseline demographic, clinical, and laboratory data were obtained within 1 week before PD catheterization. Complete blood cell count including white blood count, hemoglobin, platelets, neutrophils, lymphocytes, and monocytes were assessed. We also collected the following parameters: serum creatine, blood urea nitrogen (BUN), RRF, uric acid, calcium, phosphate, intact parathyroid hormone (iPTH), albumin, globulin, CRP, prealbumin, total cholesterol, triglyceride, high-density lipoprotein cholesterol (HDL-C), low-density lipoprotein cholesterol (LDL-C), apolipoprotein A1 (apo-A1), apolipoprotein B (apo-B), and ejection fraction (EF). The body mass index (BMI) (kg/m^2^) is equal to body weight divided by the square of height. The mean arterial pressure (MAP) was calculated by diastolic blood pressure plus 1/3 of pulse pressure. The calculation of corrected calcium was as follows: corrected calcium = serum calcium + 0.02×(40-albumin). Blood cell count-derived inflammation indexes were calculated by following equations [13]: AISI = neutrophil count × platelet count × monocyte count/lymphocyte count; SII = neutrophil count × platelet count/lymphocyte count; SIRI = neutrophil count × monocyte count/lymphocyte count.

The primary outcome was all-cause mortality. A professional PD nurse recorded patient status at the end of study (31 July 2021). The censored data included permanent switch to HD, kidney transplantation, and recovery of kidney function.

### Statistical analyses

The continuous variables were expressed as mean ± standard deviation or median (interquartile range). The unpaired t-test was used for normal distribution data and the Mann-Whitney U test for non-normal distribution data. The categorical variables expressed as frequency (percentage) were compared by Chi-Square test. Receiver operating characteristic (ROC) analysis was conducted to estimate the optimal cutoff values of AISI, SII, and SIRI. The area under the curve (AUC) was calculated to evaluate the predictive ability of these indexes.

The Kaplan–Meier curves and log-rank test were performed to estimate cumulative survival rate. Hazard ratios (HRs) and 95% confidence intervals (CIs) were calculated by Cox proportional hazards regression analyses. A significant variable by univariate analysis was further analyzed by multivariate analysis using forward stepwise regression to identify the independent prognostic power of inflammation indexes. A two-sided *p* value less than .05 indicated statistically significant difference. All statistical analyses were performed using SPSS 24.0 software.

## Results

**Figure 1.**
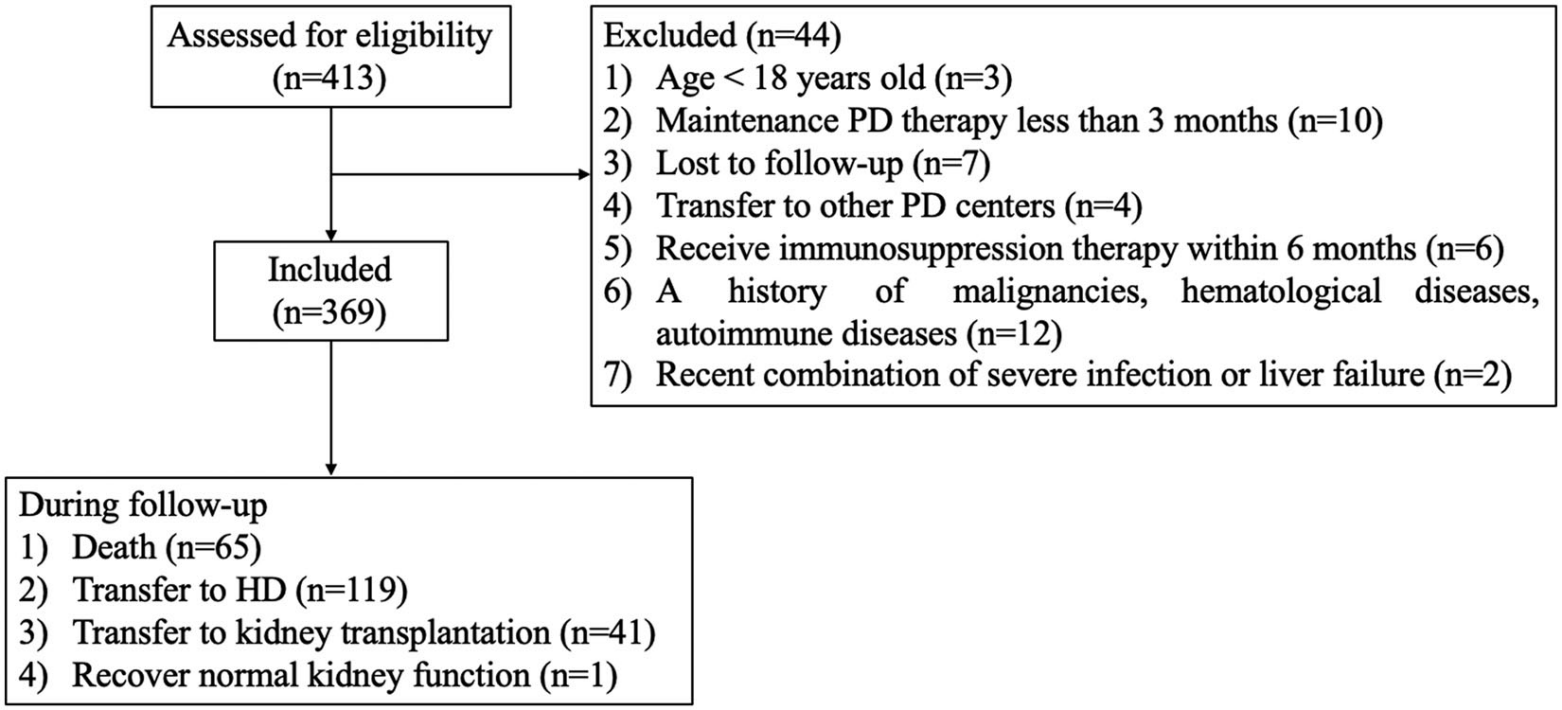
Study procedures, including patients’ selection and their outcomes.

A total of 369 incident PD patients (214 male and 155 female) were involved in this study (Figure 1). The median age was 47 (37, 59) years. Of the 369 patients, 61 (16.5%) complicated with diabetes mellitus and 41 (11.1%) had a history of cardiovascular disease (CVD). During a median follow-up period of 32.83 months, 65 patients (24.2%) died, 119 patients (44.2%) transferred to HD, 41 patients (15.2%) received kidney transplantation and 1 patient (0.4%) recovered normal kidney function. The demographic, clinical, and laboratory features are shown in Table 1. Compared with survivors, non-survivors were significantly older (*p* < .001), had higher proportions of CVD history (*p* < .001), complicated more frequently with diabetes (*p*  = .001). A significant difference in etiology of KF was also observed (*p*  = .001). Besides, non-survivors had significantly lower values of lymphocytes (*p*  = .014), higher globulin (*p*  = .005), lower serum creatinine (*p*  = .023), lower prealbumin (*p* < .001) and higher CRP level (*p*  = .012). With respect to blood cell count-derived inflammation indexes, AISI, SII, and SIRI in non-survivors were significantly higher as compared to survivors (*p*  = .003, *p* < .001, *p*  = .005, respectively). By contrast, there were no significant differences in gender, BMI, MAP, smoking status and other laboratory data between survivors and non-survivors.

**Table 1.** Demographic characteristics and laboratory data of the study population.

Clinical characteristics	All patients (*n* = 369)	Survivors (*n* = 304)	Non-survivors (*n* = 65)	*p* Value
Male (n, %)	214 (58.0%)	179 (58.9%)	35 (53.4%)	.455
Age (years)	47 (37, 59)	45 (35, 56)	63 (52, 71)	<.001
BMI (kg/m^2^)	22.50 ± 2.99	22.43 ± 3.00	22.82 ± 2.95	.348
MAP (mmHg)	111 (102, 120)	110 (102, 120)	108 (100, 120)	.325
Diabetes mellitus (n, %)	61 (16.5%)	41 (13.5%)	20 (30.8%)	.001
Current smoking (n, %)	56 (15.2%)	41 (13.5%)	15 (23.1%)	.050
CVD history (n, %)	41 (11.1%)	24 (7.9%)	17 (26.2%)	<.001
KF causes				.001
Glomerulonephritis (n, %)	205 (55.6%)	182 (59.9%)	23 (35.4%)	
Diabetic nephropathy (n, %)	34 (9.2%)	22 (7.2%)	12 (18.5%)	
Hypertension nephropathy (n, %)	36 (9.8%)	26 (8.6%)	10 (15.4%)	
Other/unknown (n, %)	94 (25.5%)	74 (24.3%)	20 (30.8%)	
White blood count (×10^9^/L)	6.07 (4.94, 7.65)	6.06 (4.93, 7.53)	6.27 (4.96, 8.22)	.293
Hemoglobin (g/dL)	8.19 (6.90, 9.30)	8.20 (6.97, 9.30)	8.03 (6.75, 9.19)	.367
Platelet count (×10^9^/L)	161 (125, 208)	159 (124, 207)	170 (137, 221)	.098
Neutrophil count (×10^9^/L)	4.23 (3.28, 5.52)	4.16 (3.25, 5.47)	4.66 (3.46, 6.63)	.061
Lymphocyte count (×10^9^/L)	1.13 (0.83, 1.53)	1.18 (0.85, 1.58)	1.02 (0.73, 1.27)	.014
Monocyte count (×10^9^/L)	0.35 (0.25, 0.48)	0.35 (0.25, 0.46)	0.40 (0.25, 0.55)	.138
AISI	205.20 (116.71, 369.63)	193.91 (106.55, 342.85)	263.19 (145.52, 626.60)	.003
SII	568.45 (371.07, 979.65)	540.84 (355.67, 874.13)	772.91 (483.89, 1300.39)	<.001
SIRI	1.2987 (0.8216, 2.0279)	1.2769 (0.7928, 1.8302)	1.6469 (1.0335, 2.9323)	.005
Albumin (g/L)	33.4 (29.8, 36.5)	33.9 (29.8, 36.5)	32.4 (28.9, 35.8)	.128
Globulin (g/L)	25.8 (22.8, 29.0)	25.6 (22.5, 28.7)	27.5 (23.8, 30.4)	.005
BUN (mmol/L)	30.50 ± 10.45	30.74 ± 10.62	29.38 ± 9.65	.343
Serum creatinine (μmol/L)	800.7 (657.1, 975.0)	811.0 (673.3, 990.6)	725.0 (625.2, 873.0)	.023
Uric acid (μmol/L)	496.02 ± 134.80	497.77 ± 138.87	487.80 ± 114.45	.589
RKF (ml/min/1.73m^2^)	5.47 (4.30, 6.97)	5.44 (4.31, 6.96)	5.52 (4.10, 7.11)	.856
Corrected calcium (mmol/L)	2.02 (1.79, 2.18)	2.02 (1.80, 2.17)	2.01 (1.72, 2.20)	.583
Phosphate (mmol/L)	1.89 (1.56, 2.34)	1.89 (1.56, 2.35)	1.89 (1.52, 2.16)	.719
iPTH (Pg/mL)	236.6 (110.3, 418.0)	239.8 (110.7, 432.8)	230.3 (98.5, 343.1)	.468
Prealbumin (mg/L)	269 (222, 304)	273 (232, 308)	232 (191, 283)	<.001
CRP (mg/L)	4.9 (3.6, 8.4)	4.6 (3.6, 7.6)	6.4 (3.7, 15.0)	.012
Cholesterol (mmol/L)	4.13 (3.49, 4.85)	4.11 (3.46, 4.81)	4.25 (3.57, 5.15)	.162
Triglyceride (mmol/L)	1.69 (1.18, 2.30)	1.70 (1.19, 2.32)	1.67 (1.12, 2.23)	.772
HDL-C (mmol/L)	0.92 (0.76, 1.12)	0.92 (0.76, 1.12)	0.95 (0.77, 1.14)	.517
LDL-C (mmol/L)	2.07 (1.65, 2.49)	2.05 (1.62, 2.48)	2.10 (1.78, 2.64)	.284
apo-A1 (g/L)	1.13 ± 0.20	1.13 ± 0.20	1.17 ± 0.20	.112
apo-B (g/L)	0.86 (0.70, 1.01)	0.85 (0.70, 1.00)	0.89 (0.75, 1.08)	.201
EF (%)	62 (59, 64)	62 (59, 64)	61 (57, 63)	.071
Follow-up time (months)	32.83 (19.63, 54.23)	32.73 (20.47, 53.93)	34.30 (17.45, 54.80)	.993

*Notes:* Continuous variables are presented as mean ± standard deviation for normal distribution data or median (interquartile range) for non-normal distribution data. BMI: body mass index; MAP: mean arterial pressure; CVD: cardiovascular disease; BUN: blood urea nitrogen; RKF: residual kidney function; iPTH: intact parathyroid hormone; CRP: C-reactive protein; HDL-C: high-density lipoprotein cholesterol; LDL-C: low-density lipoprotein cholesterol; apo-A1: apolipoprotein A1; apo-B: apolipoprotein B; EF: ejection fraction; AISI: aggregate index of systemic inflammation; SII: systemic immune-inflammation index; SIRI: systemic inflammation response index.

**Figure 2.**
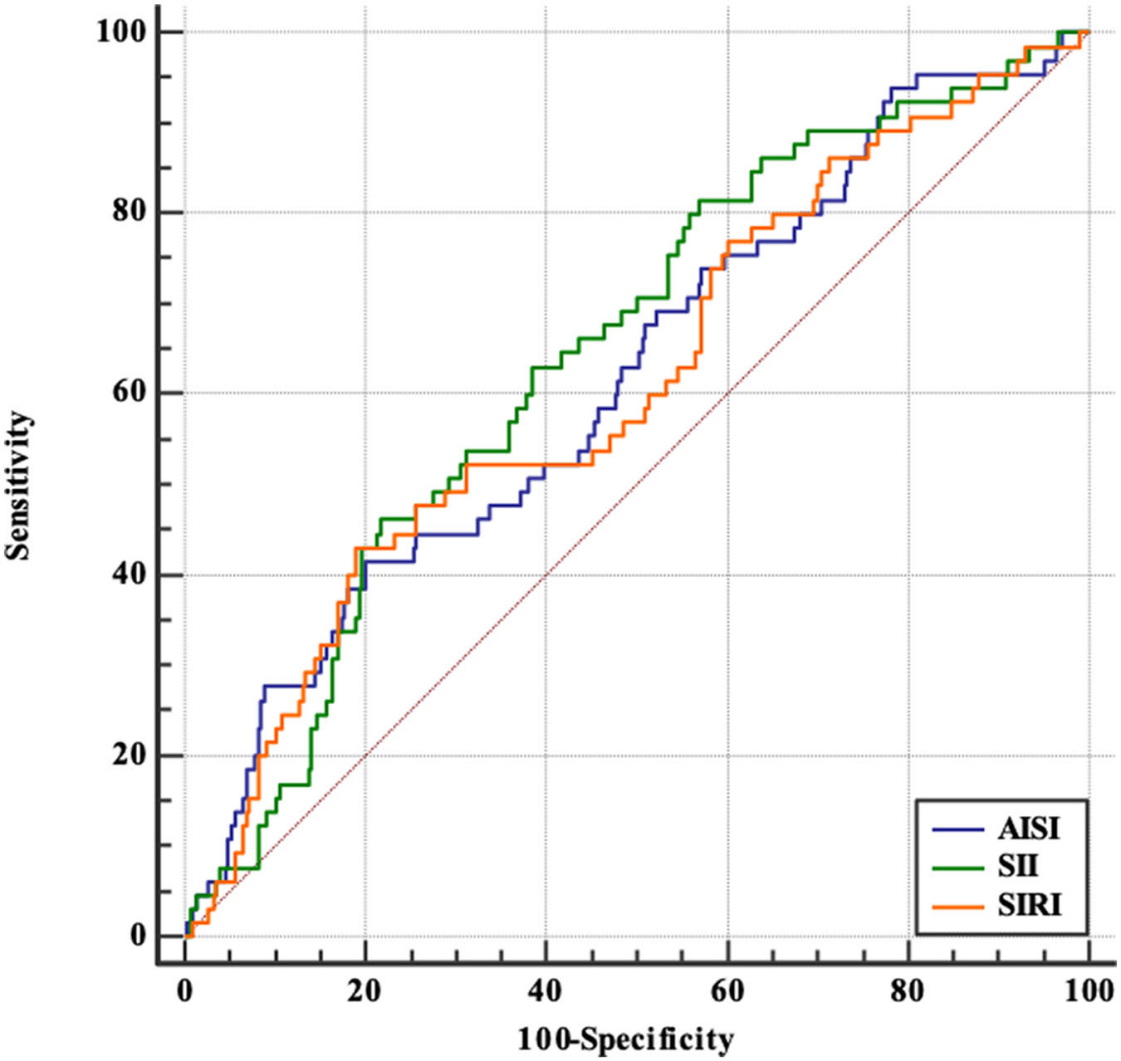
ROC curves of the probability of AISI, SII and SIRI in predicting all-cause mortality.

**Figure 3.**
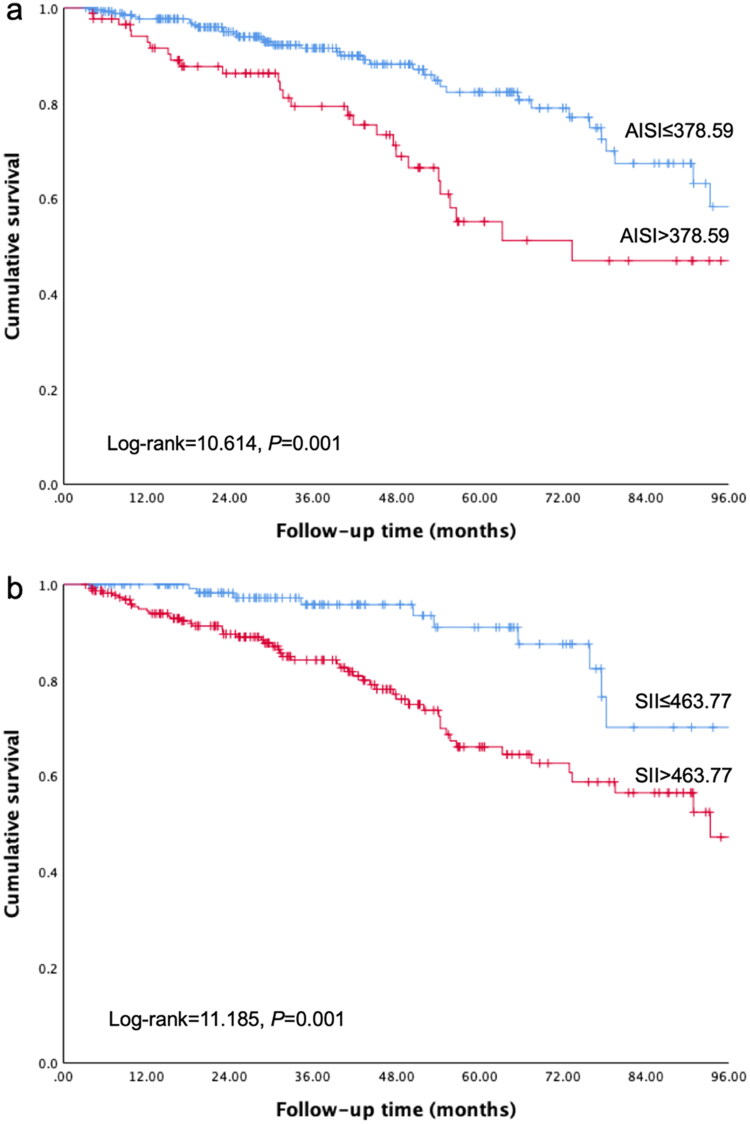

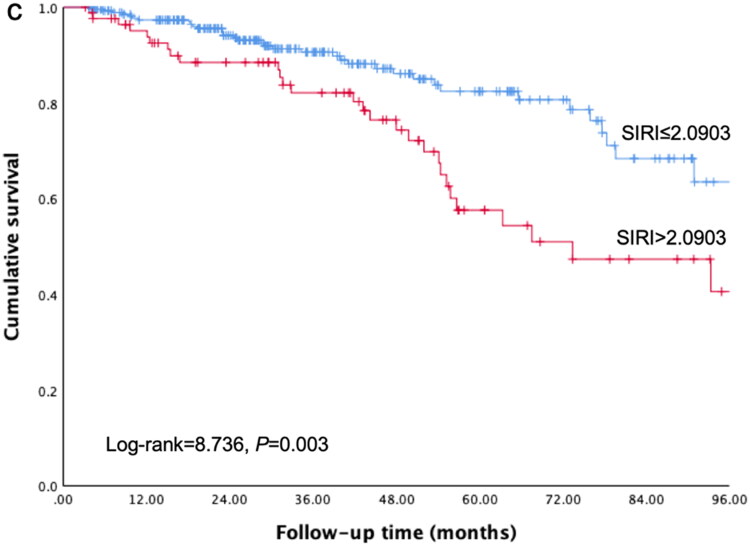
Kaplan–Meier curves for all-cause mortality in peritoneal dialysis patients with different cutoff values of the blood cell count-derived inflammation indexes. (a): AISI; (b): SII; (c): SIRI.

The ROC analysis indicated the optimal cutoff values were 378.59 for AISI, 463.77 for SII, 2.0903 for SIRI. As reported in Table 2 and Figure 2, the largest value of AUC was obtained for SII (AUC = 0.644, 95% CI = 0.573–0.715, *p* < .001), followed in order by AISI (AUC = 0.617, 95% CI = 0.541–0.693, *p*  = .003), and SIRI (AUC = 0.612, 95% CI = 0.535–0.688, *p*  = .004). The ability of CRP, NLR, PLR, and MLR to predict all-cause mortality by measuring the AUC was presented in Supplement Table 1. In contrast with CRP, the AISI, SII and SIRI showed comparable power. After classifying the patients according to cutoff values, the Kaplan–Meier survival curves revealed significantly lower survival rate with higher AISI (Figure 3(a), Log-rank = 10.614, *p*  = .001), higher SSI (Figure 3(b), Log-rank = 11.185, *p*  = .001), and higher SIRI (Figure 3(c), Log-rank = 8.736, *p*  = .003). At the end of 1, 3, and 5 years, cumulative survival rates were, respectively, 97.7%, 91.6%, and 82.3% in the low AISI group; 92.8%, 79.3%, and 55.1% in the high AISI group; 100%, 95.8%, and 91.0% in the low SII group; 94.4%, 84.2%, and 66.0% in the high SII group; 97.3%, 90.6%, and 82.4% in the low SIRI group; and 93.8%, 82.1%, and 57.6% in the high SIRI group.

**Table 2.** Optimal cutoff values and AUC of blood cell count-derived inflammation indexes.

	Cutoff value	AUC (95% CI)	*p* Value	Sensitivity (%)	Specificity (%)
AISI	>378.59	0.617 (0.541, 0.693)	.003	41.5	79.7
SII	>463.77	0.644 (0.573, 0.715)	<.001	81.5	43.1
SIRI	>2.0903	0.612 (0.535, 0.688)	.004	43.1	80.9

*Note:* AISI: aggregate index of systemic inflammation; SII: systemic immune-inflammation index; SIRI: systemic inflammation response index.

Crude Cox model analysis detected that higher AISI, SII, and SIRI were significantly associated with increased all-cause mortality (HR = 2.226, 95%CI = 1.358–3.649, *p*  = .002; HR = 2.794, 95%CI = 1.490–5.238, *p*  = .001; HR = 2.070, 95%CI = 1.264–3.389, *p*  = .004; respectively). Every increase by 1 g/dL of hemoglobin (HR = 1.040, 95% CI = 1.007–1.075, *p*  = .018) was related to 4% increased mortality risk. Other significant variables with all-cause mortality by univariate analysis is shown in Table 3. We further conducted multivariate Cox regression model analysis (Table 4). Even after adjustment for the confounding factors, higher AISI (HR = 2.508, 95% CI = 1.505–4.179, *p* < .001), SII (HR = 3.477, 95% CI = 1.785–6.775, *p* < .001), and SIRI (HR = 1.711, 95% CI = 1.012–2.895, *p*  = .045) remained as independent predictors of all-cause mortality.

**Table 3.** Univariate Cox analysis of all-cause mortality in peritoneal dialysis patients.

Variables	Univariate analysis	
	HR (95% CI)	*p* Value
Gender (female vs. male)	0.773 (0.471, 1.268)	.308
Age (≥60 years old)	4.487 (2.728, 7.379)	<.001
BMI (kg/m^2^)	1.078 (0.995, 1.167)	.065
MAP (mmHg)	0.997 (0.982, 1.013)	.735
Hypertension	3.971 (0.546, 28.881)	.173
Diabetes mellitus	3.687 (2.146, 6.335)	<.001
Current smoking	2.132 (1.195, 3.804)	.010
CVD history	3.033 (1.738, 5.292)	<.001
White blood count (×10^9^/L)	1.064 (0.961, 1.178)	.230
Hemoglobin (g/dL)	1.040 (1.007, 1.075)	.018
Platelet (×10^9^/L)	1.006 (1.003, 1.010)	.001
Neutrophil (×10^9^/L)	1.081 (0.975, 1.198)	.138
Lymphocytes (×10^9^/L)	0.736 (0.435, 1.245)	.253
Monocyte (×10^9^/L)	2.095 (0.623, 7.047)	.232
Albumin (g/L)	0.976 (0.929, 1.026)	.338
Globulin (g/L)	1.056 (1.007, 1.106)	.024
BUN (mmol/L)	0.978 (0.953, 1.004)	.101
Serum creatinine (μmol/L)	0.999 (0.998, 1.000)	.035
Uric acid (μmol/L)	0.999 (0.997, 1.001)	.161
RKF (ml/min/1.73m^2^)	1.051 (0.933, 1.183)	.413
Corrected calcium (mmol/L)	1.047 (0.507, 2.161)	.902
Phosphate (mmol/L)	0.892 (0.597, 1.333)	.577
iPTH (Pg/mL)	1.000 (0.999, 1.001)	.537
Prealbumin (mg/L)	0.992 (0.988, 0.996)	<.001
CRP (mg/L)	1.021 (1.008, 1.034)	.002
Cholesterol (mmol/L)	1.274 (1.035, 1.567)	.022
Triglyceride (mmol/L)	1.009 (0.806, 1.263)	.939
HDL-C (mmol/L)	1.439 (0.659, 3.144)	.362
LDL-C (mmol/L)	1.555 (1.156, 2.092)	.004
apo-A1 (g/L)	1.219 (0.309, 4.804)	.777
apo-B (g/L)	3.840 (1.546, 9.541)	.004
EF (%)	0.972 (0.936, 1.010)	.149
AISI > 378.59	2.226 (1.358, 3.649)	.002
SII > 463.77	2.794 (1.490, 5.238)	.001
SIRI > 2.0903	2.070 (1.264, 3.389)	.004

*Note:* BMI: body mass index; MAP: mean arterial pressure; CVD: cardiovascular disease; BUN: blood urea nitrogen; RKF: residual kidney function; iPTH: intact parathyroid hormone; CRP: C-reactive protein; HDL-C: high-density lipoprotein cholesterol; LDL-C: low-density lipoprotein cholesterol; apo-A1: apolipoprotein A1; apo-B: apolipoprotein B; EF: ejection fraction; AISI: aggregate index of systemic inflammation; SII: systemic immune-inflammation index; SIRI: systemic inflammation response index.

**Table 4.** Hazard ratios of blood cell count-derived inflammation indexes by multivariate Cox analysis of inflammation indexes.

	Model 1		Model 2		Model 3	
	HR (95% CI)	*p* Value	HR (95% CI)	*p* Value	HR (95% CI)	*p* Value
AISI > 378.59	2.330 (1.404, 3.866)	.001	2.494 (1.490, 4.175)	.001	2.508 (1.505, 4.179)	<.001
SII > 463.77	3.320 (1.691, 6.519)	<.001	2.749 (1.448, 5.221)	.002	3.477 (1.785, 6.775)	<.001
SIRI > 2.0903	1.951 (1.184, 3.215)	.009	1.852 (1.100, 3.118)	.020	1.711 (1.012, 2.895)	.045

*Notes:* Model 1: adjusted for age, diabetes mellitus, CVD history, current smoking; Model 2: adjusted for model 1 + hemoglobin, globulin, creatinine, prealbumin, CRP; Model 3: adjusted for model 2 + cholesterol, LDL-C, apo-B. AISI: aggregate index of systemic inflammation; SII: systemic immune-inflammation index; SIRI: systemic inflammation response index; CRP: C-reactive protein; CVD: cardiovascular disease; LDL-C: low-density lipoprotein cholesterol; apo-B: apolipoprotein B.

## Discussion

As far as we know, the association between the inflammation indexes (AISI, SII, SIRI) and patient survival has not been investigated in PD. Our study indicated that higher AISI, SII, and SIRI were significantly and independently correlated with all-cause mortality in PD patients, even after adjustment for confounding factors.

In our study, the cumulative survival rates were significantly lower in patients with higher AISI, SII, and SIRI. Among hematological parameters, we found significantly lower lymphocyte counts, higher AISI, SII, and SIRI in non-survivors than in survivors. Initiation of PD could either attenuate inflammation through adequate removal of uremic toxin or increase both local and systemic inflammation due to peritoneal catheter use, peritoneal fluid bio-incompatibility or PD-related infection. Accumulating evidence suggests that chronic inflammation makes a big contribution to pathogenesis and progression of chronic kidney disease (CKD) and is associated with prognosis of dialysis patients [3]. Compared with individual cell populations, indexes derived from calculating the ratios between platelets, neutrophils, monocytes and lymphocytes counts are more strongly related to chronic inflammation conditions [14]. The prognostic values of NLR, PLR, and MLR in PD patients has been investigated recently. A study included 86 PD patients and followed up for 36 months found that high NLR was a significant predictor of all-cause and cardiovascular mortality and associated with arterial stiffness [9]. In a large sample study recruiting 1652 PD patients, highest NLR tertile was correlated with increased risk of CVD events and CVD mortality in both multivariate Cox regression models and competitive risk models [15]. Another study from China detected that high PLR was an independent predictor of mortality in PD patients [10]. Elevated PLR was also found to be an independent risk factor of CVD in PD patients and overwhelmed NLR [16]. Wen et al. conducted a multicenter retrospective study including 1753 participants and suggested higher MLR levels were independently correlated with CVD mortality [11]. Based on these evidences, we speculated that AISI, SII, and SIRI calculated from more than two blood cell populations were associated with PD patient survival.

The AISI, SIRI, and SII have been proposed as novel systemic inflammation markers with prognostic relevance in patients with malignancy or undergoing major operation [17–20]. These new inflammation indexes integrate neutrophils, monocytes, lymphocytes and platelets. Neutrophils and lymphocytes are common components of these indexes. Neutrophils, the most abundant leucocytes, are the front-line cells that mediate inflammation response by migrating, producing reactive oxygen species, releasing neutrophil extracellular traps, and secreting neutrophil serine proteases [21]. In addition, neutrophils can interact with lymphocytes and monocytes through a bidirectional, multi-compartmental manner. Neutrophils recruit and activate lymphocytes and monocytes, meanwhile products of T cells and monocytes can activate neutrophils [14]. During systemic inflammation, the activation of neutrophils can stimulate the production of some mediators, such as IL-1α, which in turn stimulate megakaryocytes to produce platelets [22]. Neutrophils and monocytes participate in the persistent inflammatory response, and lymphocytes participate in immune regulation. It is acknowledged that platelet plays an important role in vascular injury and bleeding prevention. Recently, researchers suggest that platelet is capable of covering most of the stages of inflammation and a crucial mediator in response to circulating immune complexes [23]. Taken together, all of them participate in the innate and adaptive immune responses and implicate in the pathogenesis of kidney disease [24]. Consequently, an index including these populations can better reflect the inflammatory state and improve its predictive abilities.

For the first time, we found higher AISI (HR = 2.508, 95% CI = 1.505–4.179, *p* < .001), and SII (HR = 3.477, 95% CI = 1.785–6.775, *p* < .001) were still related with all-cause mortality in multivariate Cox regression models. Consistent with previous study [12], we also detected a higher SIRI (HR = 1.711, 95% CI = 1.012–2.895, *p*  = .045) corresponded to the increased all-cause mortality. The SII, calculated by multiplying platelets and NLR, has been confirmed as a prognosis indictor in COVID-19 patients [13]. Only a few studies explored the utility of SII in patients with kidney disease. A Korean cohort study including 163 patients with anti‑neutrophil cytoplasmic antibody-associated vasculitis (AAV) illustrated that high SII was correlated with increased risk of cross-sectional severe AVV, lower cumulative relapse free and kidney survivals [25]. On the contrary, a retrospective observational study conducted in Chinese patients with myeloperoxidase (MPO)‑AAV found that patients with high SII exhibited significantly higher cumulative kidney survival rates, while no significant difference in cumulative patient survival was detected. Besides, SII was positively associated with erythrocyte sedimentation rate and CRP [26]. The rational explanation for this difference is that patients with higher baseline SII showed lower serum creatinine in Chinese cohort. The severity of kidney impairment has been considered as a risk factor for kidney survival [27]. We conjectured the SII could be a potential index to reflect the inflammation response in MPO-AAV and patients with high activity index might be more likely to achieve remission. Another study indicated that SII combined with relevant clinical variables could provide useful prognostic value in patients after kidney transplantation [28]. Chen et al. [29] analyzed data of 8095 participants from Northeast China Rural Cardiovascular Health Study and implicated a linear and robust correlation between SIRI and prevalent hyperuricemia, which is closely associated with CKD [30]. Our findings provided convinced evidence that blood cell count-derived inflammation indexes could be useful predictors of PD prognosis.

In ROC curve analysis, we found the AUC of AISI, SII, and SIRI were higher than CRP (AUC = 0.599) in predicting mortality in PD patients. It is noteworthy that the predictive value was not high enough to predict prognosis based on those indexes alone. Our study demonstrated the SII provided more useful predictive value than AISI and SIRI in prognosis of PD. As a result, we proposed affordable inflammation indexes that easily derivable from routine laboratory tests and convenient for follow-up.

Several limitations should be underlined when considering our results. First, this was a Chinese single-center retrospective study with a relatively small sample size, the external validity and statistical power of our results may be restricted because of potential selection bias and center-specific effect. Hence, whether our findings could be generalizable needs future studies. Second, we appraised only baseline variables rather than longitudinal changes during follow-up, the influences of changes in the patient therapy were ignored as well. A third issue is that the optimal cutoff values of these inflammation indexes need to be further verified. Because of competing risk such as HD transfer, kidney transplantation, the incidence of all-cause mortality may potentially be inflated by Kaplan–Meier method. Future studies and evaluation should confirm the validity and accuracy of our results and establish causality between these indexes and mortality in PD patients.

## Conclusions

In summary, the present study demonstrated that the higher AISI, SII, and SIRI were independent indicators of all-cause mortality in PD patients. Furthermore, our study implicated AISI, SII, and SIRI could provide comparable predictive value. These findings point out cost-effective, widely available and simple indexes that are suitable for routine use to assist clinicians to ameliorate PD management.

## Ethics approval

The study was in line with the principles of the Declaration of Helsinki and with approved by the Ethics Committee at the Third Affiliated Hospital of Soochow University, China (registration number:19/2019).

## Consent form

All patients signed informed consent at the commencement of PD.

## Supplementary Material

Supplemental Material

## Data Availability

The data used to support the findings of this study are available from the corresponding author upon request.
