# Supplementary material for: The systemic inflammation indexes predict all-cause mortality in peritoneal dialysis patients
Source: Ren Fail. 2023 Jul 7;45(1):2160348. doi: 10.1080/0886022X.2022.2160348 (PMC10332209; doi:10.1080/0886022X.2022.2160348)
Supplement: Supplemental Material [file IRNF_A_2160348_SM6455.pdf]

Supplement Table 1. Optimal cut-off values and AUC of CRP, NLR, MLR and PLR.

	Cut-off value	AUC (95% CI)	*P*-value	Sensitivity (%)	Specificity (%)
CRP	>6.2	0.599 (0.515, 0.683)	0.021	56.9	66.4
NLR	>4.99	0.634 (0.561, 0.708)	<0.001	53.8	74.7
MLR	>0.2168	0.615 (0.542, 0.688)	0.002	90.8	30.6
PLR	>132.05	0.643 (0.571, 0.716)	<0.001	75.4	51.3

CRP: C-reactive protein; IL-6: interleukin-6; NLR: neutrophil to lymphocyte ratio; MLR: monocyte to lymphocyte ratio; PLR: platelet to lymphocyte ratio.

Information Classification: General
